# Spatial compartmentalisation of bacteria in phoronid microbiomes

**DOI:** 10.1038/s41598-023-45652-9

**Published:** 2023-10-30

**Authors:** Corey C. Holt, Sahib Dhaliwal, Ina Na, Mahara Mtawali, Vittorio Boscaro, Patrick Keeling

**Affiliations:** 1 Department of Botany, University of British Columbia, Vancouver, Canada; 2 Hakai Institute, Heriot Bay, Canada

**Keywords:** Microbiome, Molecular ecology, Water microbiology

## Abstract

The phylum Phoronida comprises filter-feeding invertebrates that live in a protective tube sometimes reinforced with particulate material from the surrounding environments. Animals with these characteristics make promising candidate hosts for symbiotic bacteria, given the constant interactions with various bacterial colonizers, yet phoronids are one of the very few animal phyla with no available microbiome data whatsoever. Here, by sequencing the V4 region of the 16S rRNA gene, we compare bacterial microbiomes in whole phoronids, including both tube and living tissues, with those associated exclusively to the isolated tube and/or the naked animal inside. We also compare these communities with those from the surrounding water. Phoronid microbiomes from specimens belonging to the same colony but collected a month apart were significantly different, and bacterial taxa previously reported in association with invertebrates and sediment were found to drive this difference. The microbiomes associated with the tubes are very similar in composition to those isolated from whole animals. However, just over half of bacteria found in whole specimens are also found both in tubes and naked specimens. In conclusion, phoronids harbour bacterial microbiomes that differ from those in the surrounding water, but the composition of those microbiomes is not stable and appears to change in the same colony over a relatively short time frame. Considering individual spatial/anatomical compartments, the phoronid tube contributes most to the whole-animal microbiome.

## Introduction

Phoronids, or horseshoe worms (phylum Phoronida), are marine invertebrates whose life cycle usually involves a pelagic larva and a benthic, sessile adult that bores into soft sediment or hard substrates like rocks or mollusc shells^1^. Phoronids filter-feed using an arch of tentacles, the lophophore, and protect their vermiform trunk with a chitinous tube excreted by epidermal glands, sometimes enriched with particulate material from its surroundings^1^. The phylum contains very few described species, but it occupies a still debated phylogenetic position within Lophotrochozoa—a key group for understanding the evolution of protostome and deuterostome metazoans^2^.

Recent efforts have dramatically expanded the number of invertebrate phyla targeted for microbiome analysis and highlighted the importance of environmental factors in shaping animal-associated bacterial community composition^3,4^. In general, the effects of host taxonomy appear to be minimal in these phyla, and relatively few bacterial sequence variants seem to be truly host-specific^3,5^. Environmental conditions like water temperature^6^ and seasonality^7^ are also known to affect the microbiome of larger filter-feeders like bivalves^8^, which show variation between tissues of the same individuals^9,10^. Bacteria isolated from the environment can also have direct implications on planktonic larvae, including those of phoronids, serving as “ecological ushers” by inducing substrate-specific metamorphosis^11^. Within phoronids themselves, there are isolated reports of bacteria inside the lophophore^12^, in-between microvilli of the body wall^13^, associated with specific layers of the tube^14^, and within specialised nerve cells in larvae^15^. However, the identities and functions of these bacteria remain unknown. Standing out against a fast-growing database of marine invertebrate microbiomes, Phoronida remains one of the very few animal phyla whose complete microbiome has never been sequenced.

**Figure 1:**
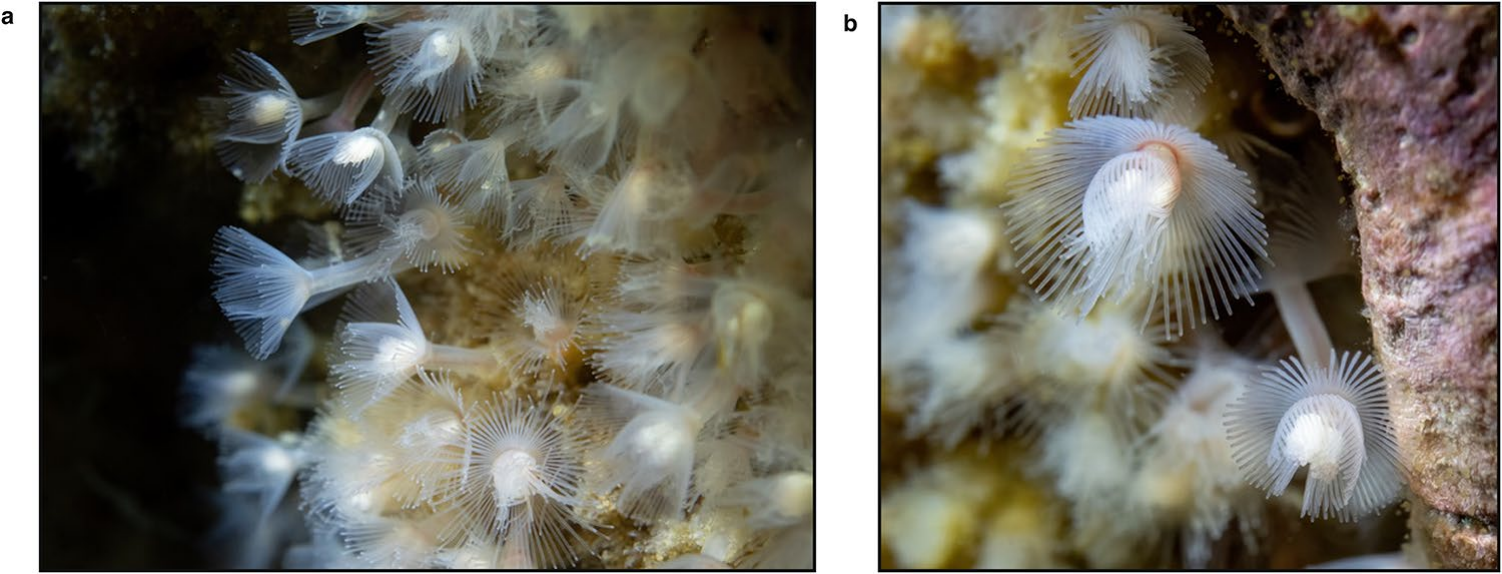
Macro images of the investigated phoronid colony in situ. (**a**) A phoronid colony showing individual animals with entangled sediment-containing tubes. (**b**). Close-up showing the distinctive horseshoe-shaped lophophore made up of feeding tentacles.

Here, we report the bacterial microbiome of phoronids collected from two consecutive months at the same location in British Columbia, Canada (Fig. 1a,b). Bacterial 16S rRNA gene libraries were generated from whole individuals, naked animals (i.e., without the external chitinous tube), and isolated tubes, to determine the importance of spatial/anatomical compartmentalisation in overall microbiome composition, within the context of community changes over time.

### Phoronid identification and lack of a eukaryotic microbiome

The phoronid 18S rRNA gene sequence obtained shows the highest sequence identities with NCBI-deposited references annotated as *Phoronis vancouverensis* (99.65–99.88%) and *Phoronis ijimai* (99.53%). Whether these species are synonymous is unclear, and the historic reliance on morphological features to determine taxonomic identity, together with a lack of molecular information from this group, has generally hindered this debate. It is noted, however, that the sequence divergence between the *P. vancouverensis* and *P. ijimai* COI gene is much lower than the range typically discriminating other phoronid species^16^.

The amplification of non-metazoan 18S rRNA gene fragments (following methods detailed in^17^) proved unsuccessful. Moreover, visual inspection through dissected tissues of at least five specimens provided no evidence of symbiotic protists. We therefore conclude that the animals in the investigated colony do not possess a significant and detectable eukaryotic microbiome. However, small marine invertebrates which often harbour symbiotic protists tend to exhibit low richness of eukaryotic Amplicon Sequence Variants (ASVs)^17^.

### Sampling date and anatomical compartment influence phoronid microbiome composition

**Figure 2:**
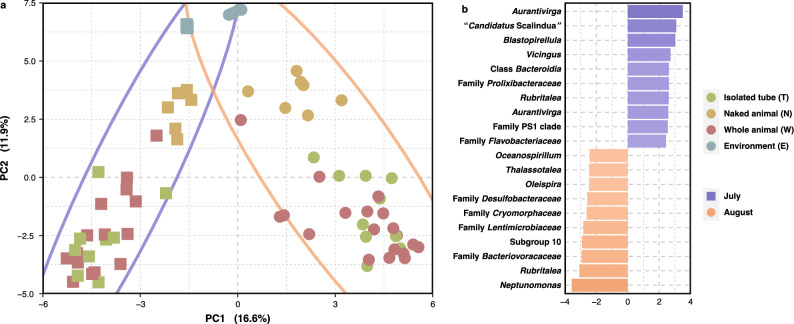
Microbiome composition differs between sampling dates. (**a**). Principal Coordinates Analysis of phoronid and environmental microbiome libraries using centred log-ratios (Aitchison distance) showing the separation into two distinct groups. Square points and purple ellipsis show July samples while circle points and orange ellipsis show August samples. (**b**) Bacterial sequence variants (labelled with the least inclusive taxon affiliation available) with the 10 largest PERMANOVA coefficients contributing to the separation of each sampling date.

Only 37.6% of bacterial variants were shared between individuals collected at different dates, and samples clustered accordingly, forming two distinct groups in an ordination using Aitchison distance (Fig. 2a; PERMANOVA: *p* value =  < 0.01; BETADISPER: *p* value = 0.078). Considering ASVs with the largest coefficients contributing to the statistical difference, *Aurantivirga, “Candidatus* Scalindua” and *Blastopirellula* ASVs are enriched in July samples, whereas *Neptunomonas, Rubritalea* and a *Bacteriovoracaceae* contribute most to August samples (Fig. 2b). Despite being common environmental taxa, all but three of the most influential ASVs determining temporal separation (*Aurantivirga*, *Rubritalea* and an unknown *Flavobacteriaceae*) were found in the phoronids themselves and not the surrounding water, as one might expect. Several of these taxa have been noted to colonise biotic surfaces^18^ or have been isolated from marine sediment^19^ and invertebrates^20^*. Aurantivirga* abundance correlates with cyclical proliferation of diatoms in phytoplankton blooms^21^ and might hence reflect environmental changes. Indeed, phoronids are known to feed on bloom-forming plankton such as diatoms and dinoflagellates^22^, therefore the phoronid microbiome is potentially highly susceptible to temporal fluctuations reflecting seasonal changes in prey abundance. “*Candidatus* Scalindua”, on the other hand, is suggested to be involved in anammox reactions in oxygen-depleted sponge tissues, so may have a more direct relationship with the phoronid host^23^. Bacterial communities in phoronid samples are significantly different from those in the surrounding water (PERMANOVA: *p* value  < 0.01; BETADISPER: *p* value =  < 0.001), which suggests that phoronids do, like other small marine invertebrates^3^, harbour a distinct bacterial microbiome that is not just a reflection of its environment.

Pairwise comparisons of microbiomes from different phoronid compartments show similar patterns in both collection dates: all comparisons between whole animals (W), naked animals (N), and isolated tubes (T) are significantly different (*p* value < 0.01), with the exception of whole animals vs. isolated tubes (July—PERMANOVA: *p* value = 0.614, BETADISPER: 0.069. August—PERMANOVA: *p* value = 0.248, BETADISPER: 0.690). A similar pattern is also reflected in pairwise Tukey comparisons of Shannon’s Diversity estimates (W vs. T, July—ANOVA: *p* value = 0.596. W vs. T, August—ANOVA: *p* value  = 0.721. All other comparisons:* p* value  < 0.05; Supplementary Table 1). This strongly suggests that, in terms of anatomical compartments, the tube has the largest influence on overall microbiome composition in phoronids, since its bacterial community is not significantly different from that observed in the whole animal.

### Phoronid-associated bacteria are often found in both the tube and the naked animal

**Figure 3:**
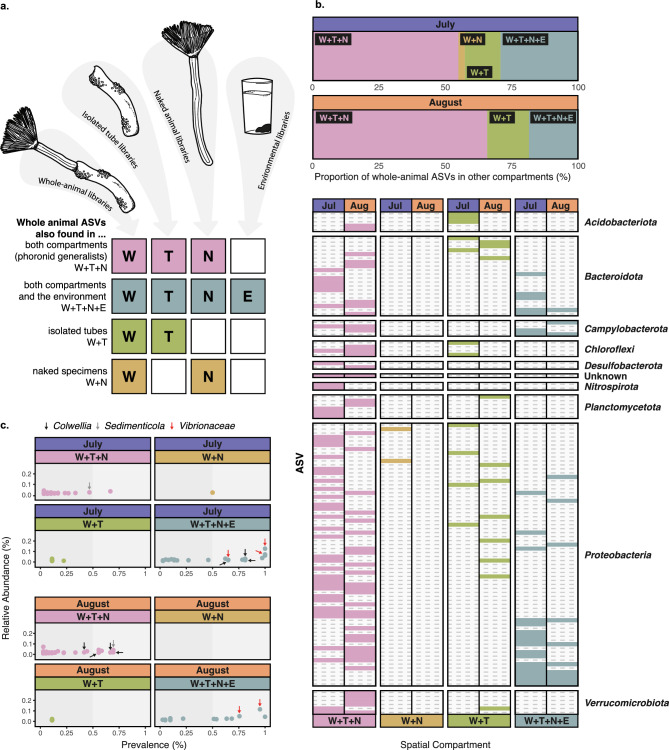
Bacterial ASVs associated with whole-animal samples that are also found in other phoronid compartments and in the environment. (**a**) Visual guideline to the colours and symbols used in the entire panel. Colours correspond to each combination of sequencing library/compartment type, showing overlap with whole-animal ASVs. Pink (generalist bacteria) = ASVs found in whole animals + isolated tubes + naked specimens (W + T + N). Aqua = ASVs found in all library types, including environment (W + T + N + E). Green = ASVs found in whole animals + isolated tubes, but not naked specimens (W + T). Gold = ASVs found in whole animals + naked specimens, but not tubes (W + N). (**b**) Top panel: bar chart showing proportion of whole-animal ASVs that are also found in other phoronid compartments from July (purple) and August (orange). Bottom panel: presence/absence of whole-animal ASVs in other library/compartment types, using the categories described in (**a**). Bacterial ASVs on the y-axis are split into phyla. (**c**) Relative abundance and prevalence of bacterial ASVs in each library/compartment type (using the categories described in (**a**). Prevalence above 50% of individuals indicated by light grey box. Black arrows show *Colwellia* ASVs, grey arrows show *Sedimenticola* ASVs, and red arrows show *Vibrionaceae* ASVs (above 25% prevalence)*.* All data in the figure is presented separately for the two collection dates.

To corroborate the impact of different animal compartments on the overall bacterial microbiome, we compared occurrences of ASVs detected in whole animals in libraries from naked specimens, the isolated tube, and the environment (Fig. 3a). Just over half of bacterial sequence variants from whole-animal libraries are also found in both isolated compartments (July: 54.9%, August: 65.7%); we refer to these taxa, belonging to different bacterial phyla, as ‘phoronid generalists’ (W + T + N in Fig. 3a,b). Prevalent generalists include the psychrophilic heterotroph *Colwellia* (also found in environmental samples from July)*,* and the sulfur-oxidizing *Sedimenticola* (Fig. 3c); the latter of which falls within a clade of several marine bivalve symbionts^24^.

Approximately 29.3% (July) and 18.6% (August) of whole-animal-associated ASVs were also found in the surrounding water (W + T + N + E in Fig. 3b), which is in line with previous small invertebrate vs. environment comparisons^3^. Bacteria in this category belonged to *Bacteroidota*, *Campylobacterota* or *Proteobacteria* and bacterial variants belonging to *Vibrionaceae* were among the most abundant in all anatomical compartments as well as water (Fig. 3c). This is likely a reflection on filter-feeding and should not be interpreted as true symbioses (although *Vibrionaceae* ASVs have been observed in other marine invertebrate microbiomes^25,26^).

Bacterial variants detected in whole-animal and tube-only samples, but absent in naked specimens, were much more prevalent than those shared with naked specimens but absent in isolated tubes (13.4 and 15.7% for W + T vs. 2.4 and 0% for W + N in July and August, respectively; Fig. 3b). Together, these results confirm that the phoronid tube is the main contributing factor impacting overall microbiome composition, although many phoronid ASVs are not strictly limited to spatial niches throughout the body plan.

## Conclusions

This first characterization of phoronid microbiomes suggests that these small animals do not harbour a substantial community of eukaryotic symbionts. They do however harbour bacterial communities partially distinct from the environmental background. Phoronid microbiome composition appears to be primarily influenced by bacteria associated with their external tube. However, bacterial sequence variants isolated from whole animals are often found in both the tube and animal tissues.

## Materials and methods

### Sample collection and processing

Animal samples were obtained from a floating dock in Whaletown Bay on Cortes Island, British Columbia, on July 11 and August 12, 2021. Small fragments (approximately 10 × 5 × 3 cm) of the substrate (inorganic flotation foam) in which the phoronid colony was embedded were collected and colony fragments with the live specimens were maintained in chilled and aerated containers of seawater collected in situ for transportation to the laboratory. Eighty-two specimens were detached from the substrate using sterile dissection tools. Forty-three of these were stored as “whole specimens” (including tissues as well as the chitinous tube) in 70% ethanol. Twenty-one isolated tubes and 39 naked animals were also collected by separating the chitinous tubes from the trunk of the animals using sterile dissection tools. Additionally, 5 phoronids from the same fragments were dissected and inspected using a Leica DM IL LED inverted microscope for the presence of host-associated protists. Animals were imaged in their natural state using a Sony A7rIII with a 50 mm macro lens and a Nauticam underwater housing with two Backscatter MW-4300 lights.

Three 150 ml water samples were collected for each colony, taking water surrounding the animals (originating from the sampling site). Each water sample was passed through a 0.8 μm GF/F Glass Microfiber Filter and the filters were immediately frozen at -80 °C until extraction.

### DNA extraction and library preparation

Total genomic DNA was extracted from animal and water samples with the QIAGEN QiaAMP DNA Mini kit. An almost full-length phoronid 18S rRNA gene sequence was obtained from one specimen extracted with the QIAGEN PowerBiofilm Kit, amplified with universal eukaryotic primers A (forward, 5′-AACCTGGTTGATCCTGCCAGT-3′) and B (reverse, 5′-TGATCCTTCYGCAGGTTCACCTAC-3′)^27^, and sent to GENEWIZ for Sanger sequencing using the same primers. Shorter 18S sequences were obtained from 16 other specimens for confirmation. Attempts to amplify non-metazoan 18S gene sequences from the extracted DNA failed, and 18S libraries obtained with universal eukaryotic primers provided exclusively phoronid sequences (data not shown). Normalized aliquots of the genomic DNA were sent to CGEB-Integrated Microbiome Resource in order to sequence the V4 region of the bacterial 16S rRNA gene on the Illumina MiSeq platform (2 X 300 paired-end sequencing).

### Bioinformatics

ASVs were generated in R^28^ with the DADA2 package (v.1.14.1)^29^ as described in^3^. Briefly, primer sequences were removed using Cutadapt (v.3.4)^30^ and reads were truncated and filtered according to their quality profiles and DADA2 standard filtering parameters (maxN = 0, maxEE = c(2,2), truncQ = 2). Error rates were characterized using the first 100 million bases and libraries were inferred with ‘pseudo’ pooling. Paired-end reads were then merged and used to generate a run-specific error model before chimaera removal and taxonomic classification against the SILVA database (v.138) with the RDP Naive Bayesian Classifier^31^. The resulting ASV table and taxonomic assignments were combined with library metadata using the phyloseq package (v.1.36.0)^32^.

The decontam package (v 1.14.0)^33^ was used to remove potential contaminants using the ‘prevalence’ method and a threshold of 0.5—meaning all sequences more prevalent in negative controls compared to true samples will be considered a contaminant. Eukaryotic, chloroplast and mitochondrial sequences were subsequently removed from the dataset, as were ASVs with a read count of 0 after library filtering. Libraries with < 1,000 reads were removed from further analysis.

ASVs with a total relative abundance < 0.001% in all libraries were removed prior to transforming read counts to centred-log ratios, and ordinating samples with an unconstrained redundancy analysis (the equivalent of a Principal Component Analysis) using Euclidean distance. The adonis function of the vegan package was used to compute a series of PERMANOVAs (using 1000 permutations)^34^, and to obtain the most significant coefficients driving the statistical difference between colonies. Pairwise PERMANOVAs were computed with the pairwiseAdonis package^35^. Homogeneity of group dispersions were tested with the betadisper function from the vegan package using the spatial median analysis^34^, and the TukeyHSD function was used for pairwise comparisons^28^. Given the statistical difference between microbiomes from different collection dates, all further statistical analyses were performed separately.

ASV richness and Shannon’s Diversity Index were generated using the estimate_richness function from the phyloseq package^32^. Pairwise comparisons were tested using TukeyHSD on a linear model generated with the aov function from base R stats package^28^. August Shannon Index data were (cube) transformed before model fitting. Good’s coverage was estimated using goods function from QsRutils^36^.

To source whole-animal occurring ASVs in different anatomical compartments, all ASVs counts were first transformed to relative abundances (%) and filtered to remove those below 1% and only consider those that were found in whole animal libraries. Three ASVs found in whole animal libraries alone were removed prior to visualisation. Seed value for all functions or plots involving random objects was set to 2209.

### Supplementary Information

Supplementary Table 1.

## Data Availability

The datasets generated during and/or analysed during the current study are available in the NCBI Sequence Read Archive (SRA) repository under the BioProject accession number PRJNA927111.
